# 4322 Structure-guided design of the TIL1383I T cell receptor

**DOI:** 10.1017/cts.2020.93

**Published:** 2020-07-29

**Authors:** Jesus Alonso, Nishant Singh, Jason Devlin, Lauren Davancaze, Brian Baker

**Affiliations:** 1 University of Notre Dame

## Abstract

OBJECTIVES/GOALS: Our goal is to employ a structure-guided design approach to engineering a safer and more effective variant of the TIL1383I T cell receptor (TCR) currently under study in clinical trials for malignant melanoma METHODS/STUDY POPULATION: Using our unpublished structure of TIL1383I we are in process of designing a panel of TCR variants with the goal of identifying candidates that improve “focus” towards the tyrosinase antigen presented on the MHC class I molecule HLA-A2. RESULTS/ANTICIPATED RESULTS: Structural analysis of TIL1383I revealed key residues, particularly beta-chain residues E97, G101, L102, responsible for engaging the tyrosinase peptide bound to HLA-A2. The crystal structure of TIL1383I in complex with tyrosinase-HLA-A2 also highlighted its uncharacteristic binding geometry and we therefore hypothesize that this binding orientation is associated with the observed CD8 co-receptor independence of TIL1383I. Indeed, functional analysis with TIL1383I-transduced CD8-positive and CD8-negative T cells, transduced T cells expressing a truncated CD8 lacking the intracellular LCK signaling domain, and tyrosinase peptide variants presented by HLA-A2 mutants outline this co-receptor independence. Combined with our interrogation of tyrosinase peptide cross-reactivity via a peptide positional scanning library approach, structure-guided design resulted in the identification of TIL1383I variants with improved binding affinities to the tyrosinase peptide as well as an understanding of structural characteristics that may contribute to TIL1383I’s co-receptor independence.

